# The Notch and TGF-β Signaling Pathways Contribute to the Aggressiveness of Clear Cell Renal Cell Carcinoma

**DOI:** 10.1371/journal.pone.0023057

**Published:** 2011-08-03

**Authors:** Jonas Sjölund, Anna-Karin Boström, David Lindgren, Sugata Manna, Aristidis Moustakas, Börje Ljungberg, Martin Johansson, Erik Fredlund, Håkan Axelson

**Affiliations:** 1 Department of Laboratory Medicine, Center for Molecular Pathology, Lund University, University Hospital MAS, Malmö, Sweden; 2 Ludwig Institute for Cancer Research, Biomedical Center, Uppsala University, Uppsala, Sweden; 3 Department of Surgical and Perioperative Sciences, Urology and Andrology, Umeå University, Umeå, Sweden; Virginia Commonwealth University, United States of America

## Abstract

**Background:**

Despite recent progress, therapy for metastatic clear cell renal cell carcinoma (CCRCC) is still inadequate. Dysregulated Notch signaling in CCRCC contributes to tumor growth, but the full spectrum of downstream processes regulated by Notch in this tumor form is unknown.

**Methodology/Principal Findings:**

We show that inhibition of endogenous Notch signaling modulates TGF-β dependent gene regulation in CCRCC cells. Analysis of gene expression data representing 176 CCRCCs showed that elevated TGF-β pathway activity correlated significantly with shortened disease specific survival (log-rank test, p = 0.006) and patients with metastatic disease showed a significantly elevated TGF-β signaling activity (two-sided Student's t-test, p = 0.044). Inhibition of Notch signaling led to attenuation of both basal and TGF-β1 induced TGF-β signaling in CCRCC cells, including an extensive set of genes known to be involved in migration and invasion. Functional analyses revealed that Notch inhibition decreased the migratory and invasive capacity of CCRCC cells.

**Conclusion:**

An extensive cross-talk between the Notch and TGF-β signaling cascades is present in CCRCC and the functional properties of these two pathways are associated with the aggressiveness of this disease.

## Introduction

Clear cell renal cell carcinoma (CCRCC) is the most common malignancy of the kidney [1]. About a quarter of the CCRCC patients have metastatic disease at the time of diagnosis and eventually one-third of the patients presented with localized tumors at diagnosis relapse. Despite recent advances using multi-kinase inhibitors, disseminated CCRCC remains inherently treatment resistant [2]. Consequently, studies leading to a better understanding of the factors that determines the metastatic phenotype of CCRCC are warranted [3]. The tumor suppressor gene *VHL* is lost in approximately 80% of all CCRCCs and represents a hallmark feature of CCRCC, but additional oncogenic events are required for both tumor formation and progression [4].

Notch signaling is an evolutionarily conserved signaling pathway of fundamental importance during development and post-natal life, regulating cell fate decisions, proliferation and survival. Dysregulated Notch signaling has been implicated in a wide variety of pathological conditions, including cancer [5]. Ligand (Jagged and Delta-like families) binding leads to two proteolytic cleavages of the receptors, the latter being dependent on the γ-secretase complex. Upon cleavage, the intracellular domain of the Notch receptor (icNotch) translocates to the nucleus where it converts the transcriptional repressor CSL to an activator [6]. Small molecule inhibitors that are capable of inhibiting Notch activation by targeting the γ-secretase complex are being tested for treatment of tumor types characterized by elevated Notch signaling, such as breast cancer and T-ALL [7]. In a recent study, we showed that Notch signaling components were elevated in primary CCRCC specimens compared to normal kidney and inhibition of Notch signaling attenuated growth of CCRCC cells, both *in vitro* and *in vivo* [8]. Thus, we have postulated that Notch signaling might represent a novel, clinically targetable oncogenic pathway in this pathological context.

The TGF-β pathway has a dual role in tumorigenesis: the growth inhibiting function at early stages of tumor formation is breached during tumor progression and at later stages TGF-β signaling can promote cell migration and invasion [9]. TGF-β elicits its cellular responses by binding to TGF-β type I and type II serine/threonine kinase receptors (TGFBR1 and TGFRBR2) that phosphorylate intracellular messengers SMAD2 and SMAD3, which in complex with SMAD4 transcriptionally induce or repress a diverse array of genes. In CCRCC, loss of TGFBR2 has been reported, which has been associated with tumor progression and also suggested to be the mechanism responsible for the escape from TGF-β-mediated growth repression [10], [11], [12], [13]. However, there are also studies showing that loss of TGFBR2 expression is associated with improved CCRCC patient survival and that the TGF-β cascade promotes CCRCC bone metastasis *in vivo* [14], [15].

Here we sought to identify downstream targets of the Notch pathway in CCRCC by employing transcriptome analyses of γ-secretase treated CCRCC cells. Our data indicate that inhibition of Notch signaling attenuates the TGF-β transcriptional output and that elevated TGF-β signaling activity in primary CCRCC is associated with decreased survival. This study thus provides additional rationale for targeting the Notch pathway for treatment of CCRCC.

## Results

### Notch inhibition in CCRCC cells affects TGF-β gene signatures

**Figure 1:**
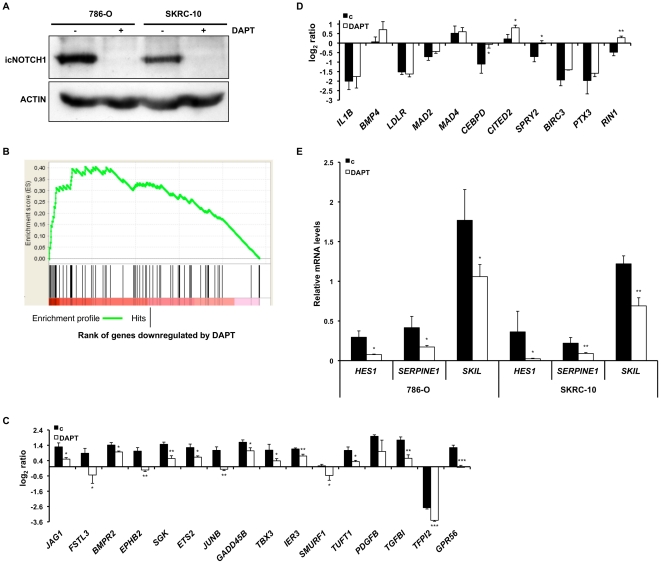
Notch inhibition in CCRCC cells attenuates TGF-β signaling. (**A**) Western blot analysis of icNotch1 in 786-O and SKRC-10 cells treated for 24 h with DAPT (+) or vehicle control (−). Cell extracts were separated by SDS-PAGE and after transfer the membrane was probed with an anti-cleaved Notch1 antibody. The blot was reprobed with an anti-ACTIN antibody to ensure equal loading. (**B**) GSEA of a TGF-β upregulated gene set classified as upregulated by TGF-β1 treatment of skin fibroblasts at 1–4 h in the SKRC-10 microarray data. Isolated and purified RNA from SKRC-10 cells, treated with DAPT or vehicle control in 1% FCS supplemented media for 24 h, was used in oligomer microarray experiments. Genes in the SKRC-10 data list were ranked for downregulation upon DAPT treatment by measure of rank product analysis FDR. Gene sets with a q-value below 0.25 were considered significantly enriched. Genes upregulated by TGF-β1 from the gene set were significantly enriched in the list ranked by downregulated by DAPT (NES = 1.75, q = 0.09). (**C**) Gene expression analysis of indicated genes reported to be activated in response to TGF-β in DAPT or vehicle control (c) treated SKRC-10 cells. Cells were treated as indicated in (**B**). Data represents mean log_2_ ratios+95% confidence intervals of three separate experiments. (**D**) Gene expression analysis of indicated genes reported to be repressed in response to TGF-β in DAPT or vehicle control (c) treated SKRC-10 cells. Cells were treated as indicated in (**B**). Data represents mean log_2_ ratios+95% confidence intervals of three separate experiments. (**E**) Notch inhibition in CCRCC cells decreases the mRNA expression of the Notch primary target *HES1* and the TGF-β target genes *SERPINE1* and *SKIL*. Q-PCR analyses of *HES1*, *SKIL* and *SERPINE1* mRNA levels in 786-O or SKRC-10 cells treated for 24 h with DAPT or vehicle control (c). mRNA levels were normalized to *SDHA*, *YWHAZ* and *UBC* expression and data represents mean+95% confidence intervals of three separate experiments. ***, ** and * indicates statistical significant changes (two-sided Student's t-test, p<0.001, p<0.01 and p<0.05 respectively).

Our previous work established that active Notch signaling is an inherent property of CCRCC cells [8]. To further confirm this observation, we performed Western blot experiments on extracts from 786-O and SKRC-10 cells using an antibody that specifically recognizes activated Notch1 (icNotch1). As anticipated, icNotch1 was detected in control treated cells whereas treatment with the γ-secretase inhibitor DAPT completely abolished the levels of icNotch1 in both cell lines (Figure 1A). We next analyzed global gene expression changes following Notch inhibition using microarrays. The Notch target genes *HES1* and *IL7R* [8], [16] were both strongly downregulated in both 786-O and SKRC-10 cells (Table 1), thus validating our approach.

**Table 1:** Top downregulated genes common to both 786-O and SKRC-10 cells upon γ-secretase inhibition when compared to vehicle treatment for 24 hours as evaluated from microarray data.

	786-O		SKRC-10	
Gene name	Mean ratio	Z	Mean ratio	Z
*HES1*	−1.44	−4.80	−2.98	−8.54
*IL7R*	−0.77	−2.57	−1.74	−5.02
*LOC284422*	−2.10	−6.96	−1.48	−4.30
*CXCL12*	−1.32	−4.38	−1.31	−3.80
*CXCR7*	−1.03	−3.44	−1.30	−3.78
***SNF1LK***	−0.71	−2.37	−1.29	−3.74
*GPR56*	−0.82	−2.73	−1.25	−3.63
*MAOB*	−1.15	−3.84	−1.22	−3.55
***SERPINE1***	−0.96	−3.19	−1.21	−3.53
*SEMA3F*	−1.11	−3.69	−1.19	−3.47
*KRTAP9-4*	−1.07	−3.59	−1.16	−3.38
*LPHN1*	−1.37	−4.58	−1.14	−3.34
*HSF2BP*	−0.71	−2.38	−1.13	−3.29
*CLTCL1*	−1.05	−3.49	−1.07	−3.13
***MN1***	−0.93	−3.10	−1.07	−3.13
*GALNT2*	−0.82	−2.76	−0.91	−2.68
*LRP4*	−0.71	−2.40	−0.90	−2.63
*CHAC1*	−0.70	−2.34	−0.84	−2.47
*FAM20C*	−0.63	−2.13	−0.80	−2.35
***INHBA***	−1.36	−4.54	−0.79	−2.32
*SR-A1*	−0.61	−2.06	−0.74	−2.18
***SKIL***	−0.68	−2.27	−0.69	−2.05

Genes in bold represents previously described TGF-β signaling target genes. Data represents mean log_2_ ratios (DAPT/DMSO) and Z-scores of three separate experiments. The genes are sorted according to the Z-scores of SKRC-10.

Interestingly, five of the most downregulated genes common to both cell lines are known TGF-β target genes [9], [17], [18] (Table 1). We next asked whether this cross talk could be statistically verified in our data using gene set enrichment analysis (GSEA) [19]. DAPT modulated gene expression in the SKRC-10 microarray experiment were ranked based on Rank product analysis FDR [20]. As exemplified in Figure 1B, GSEA showed significant enrichment of several genes related to TGF-β induced transcription among the DAPT down regulated genes [21]. In a direct comparison we noted a consistent repression of several well-described TGF-β induced genes in DAPT treated samples (Figure 1C) [9], [17], [18]. Likewise, some previously characterized TGF-β downregulated genes (e.g. *CEPBD*, *CITED2*, *SPRY2*, and *RIN1*) were significantly upregulated upon γ-secretase inhibition (Figure 1D). The downregulation of *HES1* and the TGF-β target genes *SERPINE1* and *SKIL* were confirmed using Quantitative real-time PCR (Q-PCR) in both 786-O and SKRC-10 cells (Figure 1E).

Overall, these results show that Notch inhibition not only affects prototypical Notch target genes but also modulates TGF-β dependent gene regulation in CCRCC cells.

### Association between TGF-β pathway activity and prognosis in CCRCC patients

**Figure 2:**
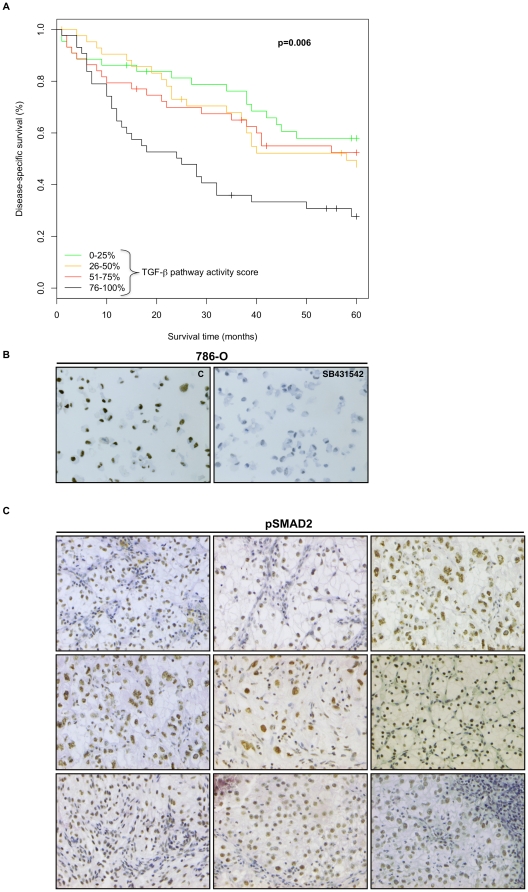
High TGF-β signaling pathway activity is associated with decreased patient survival. (**A**) Kaplan-Meier curves comparing disease-specific survival among 176 CCRCC patients divided into quartiles based on the TGF-β activity score. High TGF-β pathway activity score (76–100%) was significantly (log-rank, p = 0.006) associated with a worse disease-specific survival. (**B**) Immunohistochemical analyses using phospho-specific SMAD2 (pSMAD2) antibody of control (c) or SB431542 treated 786-O cells. Sections were counterstained with hematoxylin-eosin. Original magnification, ×40. (**C**) pSMAD2 is expressed *in vivo*. Immunohistochemistry of pSMAD2 in nine CCRCC tumors, that all showed positive nuclear staining. Sections were counterstained with hematoxylin-eosin. Original magnification, ×40.

We next investigated the clinical relevance of TGF-β signaling in primary CCRCCs. It is known that TGF-β signaling acts in a highly tissue-specific manner. We therefore extracted a core set of TGF-β target genes relevant for CCRCC cells, by analyzing published TGF-β gene expression signatures derived from hepatocytes, breast cancer and fibroblasts using GSEA [21], [22], [23]. We selected those genes from each of the TGF-β gene sets that contributed to the significant enrichment in the data from DAPT treated SKRC-10 cells, i.e. the leading edge subset from each GSEA analysis [19]. We thereby defined a core TGF-β gene expression signature of 145 genes, representing documented TGF-β target genes also affected in γ-secretase inhibited CCRCC cells (Table S1). This gene set was used to query a published gene expression data set of 176 CCRCCs [24] for correlations to survival. For each sample a specific TGF-β activity score was calculated based on the 145-gene signature. Survival analysis using Kaplan-Meier plots revealed that high TGF-β pathway activity score was significantly associated with a worse disease-specific survival (log-rank, p = 0.006; Figure 2A). Interestingly, using AJCC stage grouping, grade and performance status as covariates the TGF-β pathway activity also provided independent prognostic information when treated as a continuous variable in a multivariate Cox regression model (p = 0.004, HR = 4.04, 95% confidence interval (CI) = 1.55–10.53) (Table S2). Together, these analyses show that the TGF-β pathway is active and correlates to poor outcome in primary CCRCCs.

We next surveyed a collection of primary CCRCC specimens using an antibody directed against phosphorylated SMAD2 (pSMAD2), generally considered as a specific assessment of TGF-β signaling activity. We validated the anti-pSMAD2 antibody by staining paraffin-embedded 786-O cells that had been cultured in the presence or absence of the potent TGFBR1 inhibitor SB431542 [25] (Figure 2B). The primary CCRCCs generally showed strong nuclear pSMAD2 staining (Figure 2C), indicating that activation of the TGF-β cascade is a persistent feature of CCRCC.

### Characterization of the TGF-β responsiveness in CCRCC cells

Since the published data regarding the role of the TGF-β receptors in CCRCC are conflicting, we analyzed the prognostic impact of these receptors in the data set from 176 CCRCCs (Figure S1). Our analysis revealed a significant association between high expression of *TGFBR1* and worse disease-specific survival (log-rank, p = 0.030). Low expression of *TGFBR3* was also associated with worse disease-specific survival (log-rank, p = 0.010). No significant association between survival and expression of *TGFBR2* could be detected.

**Figure 3:**
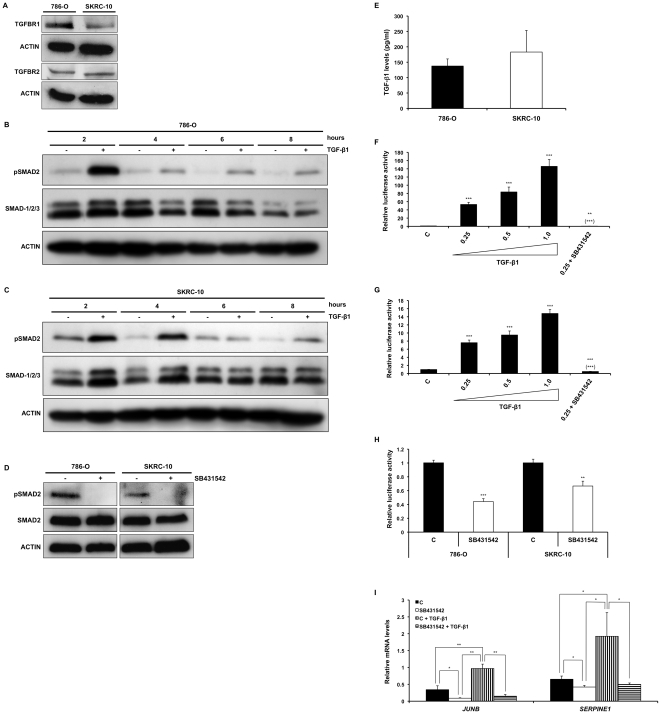
The TGF-β signaling pathway is functional in CCRCC cells. (**A**) Immunoblotting using TGFBR1 and TGFBR2 antibodies of cell lysates from 786-O and SKRC-10 cells. (**B** and **C**) pSMAD2 and SMAD-1/2/3 protein levels in cell lysates from 786-O (**B**) and SKRC-10 (**C**) cells stimulated with vehicle control (−) or 0.25 ng/ml TGF-β1 (+) for the indicated time points. (**D**) Western blot analysis using pSMAD2 antibody and SMAD2 antibody, of cell lysates from 786-O and SKRC-10 cells treated with vehicle (−) or SB431542 (+) for 4 h. (**E**) ELISA measurement of TGF-β1 levels in serum free media from 786-O and SKRC-10 cells grown for 48 h. Data represents mean+95% confidence intervals of three separate experiments. (**F** and **G**) 786-O (**F**) and SKRC-10 (**G**) cells were transfected with the SMAD regulated (*CAGA*)_12_-*Luc* reporter construct, treated with vehicle control (c), increasing concentrations of TGF-β1 or 0.25 ng/ml TGF-β1 and SB431542 for 24 h and analyzed for relative luciferase activity. Data represents mean+95% confidence intervals of three separate experiments. Data were normalized to vehicle control treated cells. (**H**) 786-O or SKRC-10 cells were transfected with the (*CAGA*)_12_-*Luc* reporter construct, treated with vehicle control (c) or SB431542 for 24 h and analyzed for relative luciferase activity. Data represents mean+95% confidence intervals of three separate experiments. Data were normalized to vehicle control treated cells. (**I**) Q-PCR analyses of *JUNB* and *SERPINE1* mRNA levels in SKRC-10 cells treated with vehicle control (c), SB431542 and/or 0.25 ng/ml TGF-β1 for 4 h. mRNA levels were normalized to *SDHA*, *YWHAZ* and *UBC* expression and data represents mean+95% confidence intervals of three separate experiments. ***, ** and * indicates statistical significant changes (two-sided Student's t-test, p<0.001, p<0.01 and p<0.05 respectively).

Studies using CCRCC cells (including the 786-O cell line) suggested that intracellular TGF-β signaling is lost due to absence of the TGFBR2 receptor [10], [11]. Baseline level of pSMAD2 could however be detected in 786-O cells using immunohistochemistry (Figure 2B). To further confirm the existence of a cell autonomous TGF-β signaling pathway in CCRCC cells, we analyzed the expression of TGFBR1 and TGFBR2 using Western blotting. Both 786-O and SKRC-10 cells expressed appreciable levels of the two receptors (Figure 3A). We also monitored the level of pSMAD2 in the presence or absence of exogenously added TGF-β1 by Western blotting. We noted a baseline activity of pSMAD2 in unstimulated 786-O and SKRC-10 cells. The level of pSMAD2 in TGF-β1 treated cells remained higher compared to control cells during the entire experiment in both cell lines (Figure 3B and 3C). Treatment with SB431542 led to a complete loss of the pSMAD2 signal (Figure 3D). Previous reports have indicated that the expression of TGF-β1 is elevated in CCRCC due to the loss of pVHL [10], [26], which are results compatible with our observations of pSMAD2 expression in primary CCRCCs and baseline expression of pSMAD2 in unstimulated CCRCC cells. In order to assess TGF-β1 production in 786-O and SKRC-10 cells, we employed an ELISA assay. After 48 hours, TGF-β1 could be readily detected in the medium of both cell lines (Figure 3E). When 786-O and SKRC-10 cells were transfected with a plasmid containing a SMAD regulated luciferase reporter ((*CAGA*)_12_-*Luc*), a dose-dependent increase of the luciferase reporter was detected upon TGF-β1 stimulation (Figure 3F and 3G, respectively). This induction was dependent of TGFBR1 activation, since treatment with SB431542 abrogated both TGF-β1 induced and basal reporter gene activity (Figure 3F–H). Q-PCR experiments showed that the expression of the TGF-β responsive genes *JUNB* and *SERPINE1* were significantly induced upon treatment with TGF-β1 and suppressed below baseline when the cells were treated with SB431542 alone or in combination with TGF-β1 (Figure 3I).

Based on these experiments, we conclude that the TGF-β signaling pathway is functional in the two investigated CCRCC cell lines and that the basal activity might be a consequence of endogenous TGF-β1 production.

### Notch inhibition perturbs both basal and induced TGF-β signaling activity

**Figure 4:**
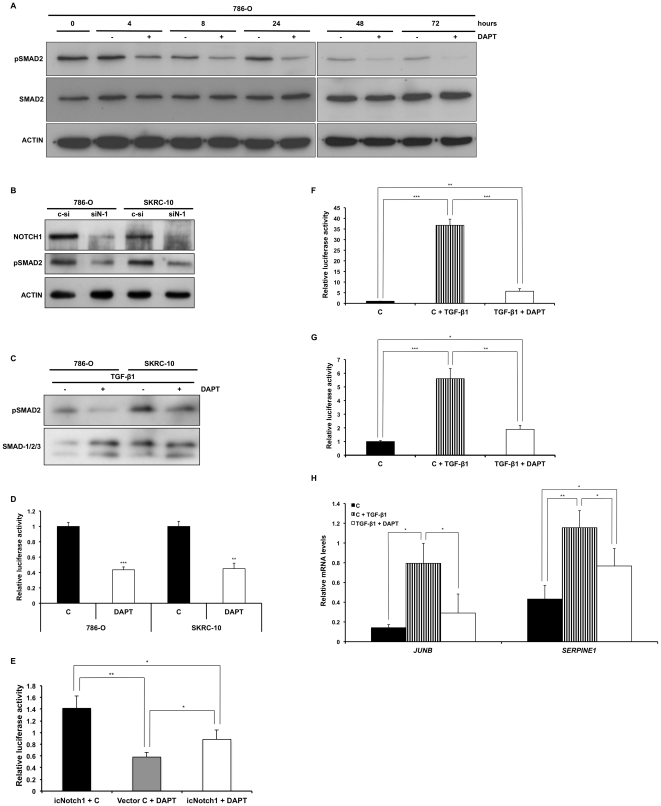
Notch inhibition alters endogenous and TGF-β1 induced activation of the TGF-β signaling pathway. (**A**) Immunoblotting using pSMAD2 antibody and SMAD2 antibody, of cell lysates from 786-O cells stimulated with vehicle control (−) or DAPT (+) for the indicated time points. (**B**) Immunoblotting using Notch1 antibody and pSMAD2 antibody of cell lysates from 786-O and SKRC-10 cells transfected with control siRNA (c-si) or siRNA against *Notch1* (siN-1). Cells were harvested after 24 h of transfection. (**C**) Western blot analysis, using pSMAD2 antibody and SMAD-1/2/3 antibody of cell lysates from 786-O and SKRC-10 cells stimulated with 0.25 ng/ml TGF-β1 and vehicle (−) or 0.25 ng/ml TGF-β1 and DAPT (+) for 4 h. (**D**) 786-O and SKRC-10 cells transfected with the (*CAGA*)_12_-*Luc* reporter construct and treated with vehicle control (c) or DAPT for 24 h and followed by measurment of relative luciferase activity. Data represents mean+95% confidence intervals of three separate experiments. Data were normalized to vehicle control treated cells. (**E**) Relative luciferase activity in extracts from 786-O cells transfected with the (*CAGA*)_12_-*Luc* reporter construct and vector control (Vector C) or icNotch1 expression vector followed by treatment with DAPT or vechicle control (c) for 24 h. Data represents mean+95% confidence intervals of three separate experiments. Data were normalized to vehicle control treated and vector control transfected cells. (**F** and **G**) 786-O (**F**) and SKRC-10 (**G**) cells were transfected with the *(CAGA)_12_-Luc* reporter construct, treated with vehicle control (c), 0.25 ng/ml TGF-β1 and vehicle control or 0.25 ng/ml TGF-β1 and DAPT for 24 h and analyzed for relative luciferase activity. Data represents mean+95% confidence intervals of three separate experiments. Data were normalized to vehicle control treated cells. (**H**) Q-PCR analyses of *JUNB* and *SERPINE1* mRNA levels in SKRC-10 cells treated with vehicle control (c), 0.25 ng/ml TGF-β1 and vehicle (c) or 0.25 ng/ml TGF-β1 and DAPT for 4 h. mRNA levels were normalized to *SDHA*, *YWHAZ* and *UBC* expression and data represents mean+95% confidence intervals of three seperate experiments. ***, ** and * indicates statistical significant changes (two-sided Student's t-test, p<0.001, p<0.01 and p<0.05 respectively).

We next wanted to characterize the effects of Notch inhibition on TGF-β signaling. 786-O cells showed a modest but consistent decrease of basal pSMAD2 levels upon treatment with DAPT at all time-points analyzed, while the SMAD2 levels were unaffected all through the time course of the experiment (Figure 4A). Similar results were obtained when analyzing the SK-RC10 cell line (data not shown). Also, when transfecting the cells with siRNA against *Notch1* a down-regulation of pSMAD2 levels could be detected (Figure 4B). Notch inhibition also decreased phosphorylation of SMAD2 in cells stimulated with TGF-β1 (Figure 4C).

We next wanted to analyze whether the suppressive effect on TGF-β pathway activity by Notch inhibition could be reversed by constitutively active, γ-secretase insensitive Notch signaling. For this purpose, we co-transfected 786-O cells with the (*CAGA*)_12_ luiferase reporter together with an icNotch1 expression vector and treated the cells with DAPT. As shown in figure 4E, expression of icNotch1 led to a significant increase in reporter activity compared to vector control. In the presence of DAPT, expression of icNotch1 led to a partial but significant reversal of the DAPT induced suppression of reporter activity (Figure 4E). We further corroborated the diminished basal and TGF-β1 induced TGF-β activity upon treatment with DAPT using the (*CAGA*)_12_ reporter (Figure 4D, 4F and 4G). Modulation of Notch signaling also affected TGF-β responsive genes (*JUNB* and *SERPINE1*) in the presence of TGF-β1 (Figure 4H).

Altogether, our data indicate that inhibition of Notch signaling downregulates TGF-β signaling in CCRCC cells.

### Notch inhibition perturbs the migratory capacity of CCRCC cells

**Figure 5:**
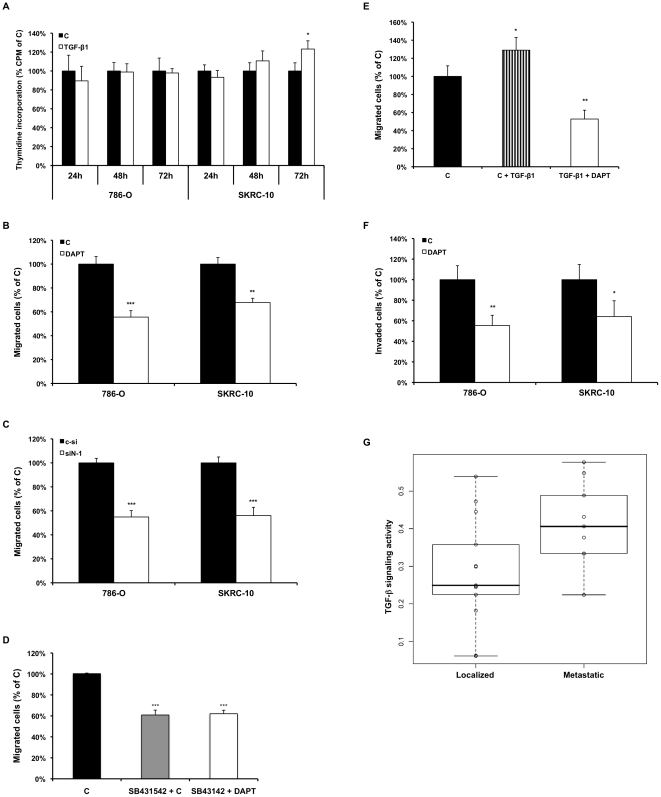
Notch inhibition attenuates CCRCC cell migration and invasion. (**A**) [^3^H]thymidine incorporation of 786-O and SKRC-10 cells grown for indicated hours (h) in the presence of 1.0 ng/ml TGF-β1 or treated with vehicle control (c). Data represents mean+95% confidence intervals of three separate experiments. Data were normalized to vehicle control treated cells. (**B**) Cell migration assessed by Boyden chamber assays of 786-O and SKRC-10 cells treated with vehicle control (c) or DAPT. The cells were allowed to migrate towards the lower compartment for 4 h (786-O) or 5 h (SKRC-10). Data represents mean+95% confidence intervals of three separate experiments. Data were normalized to vehicle control treated cells. (**C**) Cell migration as determined by Boyden chamber assays of 786-O and SKRC-10 cells transfected with non-specific control (c-si) or *Notch1* (siN-1) specific siRNAs. After 24 h of transfection, cells were counted and seeded into the Boyden chamber. The cells were allowed to migrate towards the lower compartment for 4 h (786-O) or 5 h (SKRC-10). Data represents mean+95% confidence intervals of three separate experiments. Data were normalized to vehicle control treated cells. (**D**) Cell migration as determined by Boyden chamber assays of SKRC-10 cells treated with vehicle control (c), 2 µM SB431542, 10 µM DAPT and control. The cells were allowed to migrate towards the lower compartment for 5 h. Data represents mean+95% confidence intervals of three separate experiments. Data were normalized to vehicle control treated cells. (**E**) Cell migration analyses of SKRC-10 cells treated with vehicle control (c) or treated with 0.25 ng/ml TGF-β1 and vehicle (c) or 0.25 ng/ml TGF-β1 and DAPT. The cells were allowed to migrate towards the lower compartment for 5 h. Data represents mean+95% confidence intervals of three separate experiments. Data were normalized to vehicle control treated cells. (**F**) Cell invasion as determined by Matrigel coated Boyden chamber assays of 786-O and SKRC-10 cells treated with vehicle control (c) or DAPT. The cells were allowed to invade and migrate towards the lower compartment containing 10% FCS for 16 h (786-O) or 21 h (SKRC-10). Data represents mean+95% confidence intervals of three separate experiments. Data were normalized to vehicle control treated cells. ***, ** and * indicates statistical significant changes (two-sided Student's t-test, p<0.001, p<0.01 and p<0.05 respectively). (**G**) TGF-β pathway activity correlates to metastatic spread of primary CCRCCs. CCRCCs from patients with either metastatic disease at diagnosis or that later developed metastasis (Metastatic, n = 13) showed a significantly elevated TGF-β signaling activity based on the 145-gene TGF-β signature as compared to tumors from patients with a localized disease and with no documented metastases during follow-up (Localized, n = 9) (two-sided Student's t-test, p = 0.044).

The dual role of TGF-β signaling in cancer is well established, with a cytostatic effect in the early stages, which can be subdued to a metastatic promoting program at the later stages of tumor progression [17]. We noted however very modest effects on the cytostatic TGF-β transcripts [9] in DAPT treated SKRC-10 cells (Figure S2A). Consistent with this observation and with a previous report [10], thymidine incorporation assays confirmed that the growth capacity of CCRCC cells was not decreased by treatment with TGF-β1 for up to 72 h (Figure 5A). In case of the SKRC-10 cells there was even a modest but significant increase in growth at 72 h of treatment with TGF-β1.

Our microarray experiments indicated that genes regulating migration and/or invasion were downregulated (Figure S2B). Most of these genes are also directly regulated by TGF-β signaling and have been associated with aggressive and invasive cancer [17]. This observation suggested that Notch inhibition perturbs the migratory and/or invasive capacity of CCRCC cells. We functionally verified this using Boyden chamber assays and noted a significant decrease in migration when CCRCC cells were treated with DAPT (Figure 5B) or upon *Notch1* knockdown (Figure 5C) compared to control treated cells. Also, treatment with the TGF-β inhibitor SB431542 led to a significant decrease in migration of SK-RC10 cells and when combining SB431542 and DAPT treatments, no further decrease in migration was noted (Figure 5D). Addition of exogenous TGF-β1 further stimulated the migratory capacity and this effect could be attenuated by Notch inhibition (Figure 5E). Furthermore, Notch inhibition led to a pronounced and significant decrease in invasion in both cell lines tested when compared with vehicle control (Figure 5F). To verify the clinical significance of these results, we assessed TGF-β signaling activity based on our 145-gene TGF-β signature in a previously published microarray study [27]. CCRCCs from patients with either metastatic disease at diagnosis or that later developed metastasis (n = 13) showed a significantly elevated TGF-β signaling activity as compared to tumors from patients with a localized disease and with no documented metastases during follow-up (n = 9) (two-sided Student's t-test, p = 0.044, Figure 5G).

Thus, dysregulated Notch signaling might contribute to CCRCC aggressiveness at least in part by modulating TGF-β signaling activity.

## Discussion

It has been shown that loss of VHL, which is the key oncogenic event in CCRCC, leads to elevated expression of TGF-β1 [10]. Interestingly, elevated levels of TGF-β1 in serum from CCRCC patients are correlated with unfavorable outcome of the disease [26], [28], [29]. Hence, the tumor microenvironment in CCRCC is rich in TGF-β1. These observations therefore suggest that CCRCC cells might have acquired the capacity to evade the cytostatic effects imposed by the presence of TGF-β1. It has been postulated that structural alterations of TGF-β pathway components, such as mutations of *TGFBR2* render tumor cells insensitive to TGF-β cytostatic effects [17]. In CCRCC there are conflicting reports on such alterations and there is an apparent lack of functional analyses of signaling activity, e.g. assessment of pSMAD2 levels. Experimental *in vitro* and *in vivo* studies have indicated that TGBR3 have antitumoral effects in CCRCC cells independent of TGF-β1 and canonical TGFBR1/TGFBR2/SMAD signaling [11]. Our data showing that low *TGFBR3* expression in primary CCRCC is significantly associated with worse disease-specific survival is thus adding further support for this notion. Loss of TGFBR2 has been linked to CCRCC progression [10], [11], [12], [13], whilst another investigation showed that loss of TGFBR2 improve CCRCC patient survival [15]. In favor of the latter study, the TGF-β cascade has been shown to promote CCRCC bone metastasis *in vivo* [14]. It is noteworthy that Ananth *et al*, concluded that the 786-O cells lacks a working TGF-β signaling pathway due to the absence of TGFBR2 expression [10]. In contrast, our functional assessment of the pathway in 786-O cells clearly shows that the pathway remains intact. In normal renal cells, TGF-β1 elicits an antimitogenic response and triggers epithelial-to-mesenchymal transition [18], [30], [31], [32], [33]. While our data indicate that CCRCC cells are insensitive to TGF-β-induced growth inhibition, the cells retain an operational TGF-β pathway that directs pro-migratory and pro-metastatic functions. Consistent with the experimental data, we found evidence of SMAD2 activation in clinical specimens and an association between TGF-β signaling activity, disease-specific survival and metastatic progression in the analyses of primary CCRCCs. Our observation that elevated *TGFBR1* is significantly associated with worse disease-specific survival provides further support for a pro-metastatic function of TGF-β signaling in CCRCC. Thus, we extend previous data and suggest a pro-oncogenic role for a hyperactivated autocrine TGF-β pathway in CCRCC. This tumor-promoting effect of pathogenic TGF-β signaling could partly be manifested in an increased metastatic potential of the tumor cells, but also through paracrine angiogenic and immunosuppressive effects of TGF-β secreted by the growing tumor mass [10], [34].

Different modes of cross-talk between the TGF-β and Notch signaling pathways of both synergistic and antagonistic nature have been reported in various cellular contexts [35], [36], [37], [38], [39], [40], [41], [42], [43], [44]. In CCRCC cells, characterized by high activity of both pathways, Notch signaling seems superimposed on TGF-β signaling since Notch inhibition, either by siRNA targeting *Notch1* or pharmacological inhibition of Notch receptor activation, clearly perturbs important aspects of metastasis associated TGF-β signaling.

Since metastatic CCRCC has a particularly poor prognosis, with a five-year survival of about 9%, it is critical to develop treatment strategies that target the metastatic process [1]. We have recently developed a novel γ-secretase inhibition strategy, using intermittent treatment cycles that strongly inhibited the growth of xenotransplanted CCRCC cells while limiting the toxicity of the intestine, which is a major obstacle in achieving effective doses of these drugs in humans [8]. In a recent study it was also shown that glucocorticoids abrogate the gastrointestinal toxicity of γ-secretase inhibitors [45]. Thus, these studies provide alternative strategies to spare the patients from the side effects of systemic Notch inhibition.

We now provide evidence that Notch inhibition also attenuates the migratory capacity of CCRCC cells, at least in part through modulation of TGF-β signaling. In addition, it is known that inhibition of Notch signaling perturbs tumor angiogenesis [46]. Thus, we conclude that Notch inhibition might be a particularly appealing approach for treatment of CCRCC, potentially curbing several key aspects of tumor aggressiveness.

## Materials and Methods

### Cell culture and reagents

The 786-O (ATCC, Rockville, MD, USA) CCRCC cell line was cultured in DMEM (Invitrogen, Stockholm, Sweden) containing 10% fetal calf serum (FCS, Invitrogen) and supplemented with 1% penicillin and streptomycin (PEST, Invitrogen). The SKRC-10 CCRCC cell line was maintained in RPMI 1640 (Invitrogen) containing 10% FCS and 1% PEST. Human recombinant TGF-β1 was obtained from PeproTech (London, United Kingdom). Cells were treated with 2 µM TGFBR1 inhibitor (SB431542, Sigma Aldrich, Munich, Germany), 10 µM γ-secretase inhibitor DAPT (*N*-[N-(3,5-difluorophenacetyl)-L-alanyl]-S-phenylglycine t-butyl ester) from Calbiochem (Darmstadt, Germany) or the corresponding volume of DMSO (Sigma Aldrich) for indicated times. All experiments were performed in reduced serum conditions.

### Microarray and data analyses

RNA from 786-O and SKRC-10 cells, treated with DAPT or vehicle control in 1% FCS supplemented media for 24 h, was used for gene expression microarray experiments with a 27 k cDNA array platform (http://www.lth.se/sciblu). Array production, sample labeling, hybridization and scanning were performed essentially as described previously [47]. In short, 5 µg of total RNA (DAPT or vehicle control) was labeled with Cy3 and hybridized against 5 µg of Cy5-labeled RNA from a pool representing nine untreated CCRCC cell lines. As the effects of DAPT treatment were of different magnitude in SKRC-10 and 786-O cells, a comparative Z-score was calculated by dividing the mean log_2_ ratio values for each gene and cell line with the standard deviation of all mean log_2_ ratios for each cell line. We perfomed a second round of experiments (dubbed SKRC-10 data set), that were used for GSEA [19] and extraction of gene expression signatures for pathway analysis. Rank product analysis [20] was used to create ranked gene lists based on both upregulation and downregulation. The downregulated ranked gene lists were used for correlation analyses to known gene signatures according to the GSEA method using the Molecular Signatures Database (MSigDB), and additional published TGF-β regulated gene sets [22], [23].

Genes in the SKRC-10 data set contributing to a significant enrichment of the TGF-β gene sets were thereafter used to generate a DAPT/TGF-β specific signature. To investigate possible clinical significance of this obtained TGF-β gene signature, two gene expression data sets were used. The first, which comprised 177 CCRCCs, was obtained from the Stanford microarray database [48] and normalized as described in the original publication [24]. Replicate reporters were merged by gene-symbol and a presence filter was applied allowing not less than 50% presence for each gene across arrays. The second data set comprised 22 CCRCCs and 23 normal kidney samples [27]. Log_2_ expression values for each reporter were centered according to the median expression of the normal samples and replicate reporters were merged by gene symbol. For each sample, a TGF-β pathway activity score was calculated as previously described [49]. Disease-specific survival (DSS) and American Joint Committee on Cancer (AJCC) stage grouping (I, II, III or IV) were available for 176 of the 177 patients in the Zhao *et al* cDNA gene expression data set [24]. Follow-up time was limited to five years. For Kaplan-Meier analyses, patients were divided into quartiles based on their relative TGF-β pathway activity score and interquartile differences in survival were assayed using the log-rank test. All statistical analyses were carried out using the R statistical programming environment (http://www.r-project.org). Specifically, for survival analyses the Survival package was used.

### Quantitative real-time PCR analyses

Q-PCR analyses, total RNA extraction and quantification of gene expression using SYBR Green (Applied Biosystems, Foster City, CA, USA) were done according to previously published procedures [50]. Primer sequences are given in Table S3. Quantification of relative mRNA levels was done using the comparative C^t^ method and normalized to three endogenous references genes (*SDHA*, *YWHAZ* and *UBC*) [51].

### Luciferase reporter assays and siRNA transfection

For siRNA experiments, cells were transfected with control siRNA or siRNA against *Notch1* (Santa Cruz Biotechnology, Santa Cruz, CA, USA) using Lipofectamine 2000 (Invitrogen) and OptiMEM I Reduced Serum Medium (Invitrogen) as described elsewhere [8].

For luciferase experiments, cells were transiently transfected with the luciferase reporter vector (*CAGA*)_12_-*Luc* containing 12 *CAGA* SMAD binding sites [52]. ph*RL*-*TK* renilla expression vector (Promega, Madison, WI, USA) was used as a control for transfection efficiency. The icNotch1 expression construct was kindly provided by J.C. Aster [53]. Cells were lysed and assayed for luciferase and renilla activities using the Dual-Luciferase Reporter Assay System (Promega).

### Western blot analyses and immunohistochemistry

Cells were lysed in RIPA buffer, separated on a SDS–PAGE gel and blotted onto Immobilon-P (Millipore, Bedford, MA, USA) or Hybond-C (Amersham Biosciences, Uppsala, Sweden) membranes. The membranes were incubated with the following primary antibodies: anti-Notch1 (Santa Cruz Biotechnology), anti-cleaved Notch1 (Cell Signaling Technology, MA, USA), anti-phosphorylated SMAD2 (pSMAD2, Cell Signaling Technology), anti-SMAD2 (Cell Signaling Technology), anti-SMAD-1/-2/-3 (Santa Cruz Biotechnology), anti-TGFBR1 (Santa Cruz Biotechnology), anti-TGFBR2 (Santa Cruz Biotechnology) or anti-ACTIN (ICN Biomedicals, Aurora, OH, USA). HRP-conjugated secondary antibodies were obtained from Amersham Biosciences, Dako (Glostrup, Denmark) and Jackson ImmunoResearch Laboratories Inc (West Grove, PA, USA). Proteins were detected by Super Signal chemiluminescence substrate (Pierce, Rockford, IL, USA).

Tumor samples collected at the University Hospital in Umeå, Sweden, including nine nephrectomy specimens were analyzed by immunohistochemistry. The tumors were classified as CCRCCs according to the Heidelberg classification system [54]. All tumor samples were obtained after permission from the patients with informed and signed consent, and the Institutional Review Board approved the study. pSMAD2 immunoreactivity was detected using the Dako EnVision system and the Dako TechMate 500 as previously described [8]. Sections were counterstained with hematoxylin-eosin. To evaluate antibody specificity, pSMAD2 immunoreactivity of vehicle control treated 786-O cells or 786-O cells in which the antigen had been eliminated by SB431542 for 24 h were performed.

### TGF-β1 ELISA assay

Cells were maintained in FCS-free media for 48 h, whereafter an ELISA was performed using the Human TGF-β1 immunoassay (R&D Systems Inc, Minneapolis, MN, USA) according to the manufacturer's description. An ELISA microplate reader (BioTek Synergy 2, Fisher Scientific, Gothenburg, Sweden) was used to analyze the absorbance (450 nm).

### Cell proliferation assays

Cells were seeded in 1% FCS media supplemented with vehicle control or TGF-β1 and incubated for 24, 48 or 72 h. [^3^H]thymidine (Amersham Biosciences) was then added to the culture. Cells were harvested after 24 h of incubation. The incorporated [^3^H]thymidine was measured in a ß-liquid scintillation counter (LKB RackBeta Wallace, Turku, Finland).

### Migration and invasion assays

In γ–secretase inhibition experiments, cells were pretreated for 24 h with DAPT or vehicle control before initiation of the migration assay. Cells were then seeded in FCS-free media supplemented with DMSO, DAPT and/or TGF-β1 into Boyden chambers (Corning, Bodenheim, Germany) with 8-µm pore size polycarbonate membrane filters. In experiments combining SB431542 and DAPT, SKRC-10 cells were pretreated with 2 µM SB431542 alone, in combination with 10 µM DAPT or with vehicle control (DMSO) in 1% FCS supplemented media for 24 h. The cells were then allowed to migrate towards the lower compartment containing 10% FCS for 4 h (786-O) or 5 h (SKRC-10). The migrated cells were then fixed with 4% paraformaldehyde (Sigma Aldrich) and stained with DAPI ([4′,6′-diamidino-2-phenylindole], Sigma Aldrich). The cells were thereafter counted by microscopy at 40× magnification. Four representative fields were counted for each filter, and each treatment condition was assayed in triplicate and repeated three times. In siRNA experiments, cells were transfected with control siRNA or siRNA against *Notch1* 24 h preceding migration assay.

For invasion assays, 12.5% Growth Factor Reduced BD Matrigel™ Matrix (BD Biosciences, Bedford, MA, USA) diluted in FCS-free media was added on top of each Boyden chamber membrane. The cells were seeded in FCS-free media supplemented with DAPT or DMSO and were then allowed to invade through the Matrigel towards the lower compartment containing 10% FCS for 16 (786-O) or 21 h (SKRC-10) at 37°C. After incubation cells were analyzed as described for the migration assay.

### Statistical Analysis

Data were calculated as the mean values with 95% confidence intervals. All statistical tests were two-sided Student's t-test and statistical significance was defined as p less than 0.05. For the statistical design and analyses of gene expression microarray data refer to “Microarray and data analyses” above.

## Supporting Information

Figure S1 **Disease-specific survival of 176 CCRCC patients based on the gene expression of TGFBRs.** Kaplan-Meier plots of disease-specific survival of 176 CCRCC patients that were divided into four groups based on the median gene expression values of *TGFBR1*, *TGFBR2*, and *TGFBR3*. Elevated *TGFBR1* expression (log-rank, p = 0.030) and decreased (0–25%) *TGFBR3* expression (log-rank, p = 0.010) were significantly associated with worse disease-specific survival.
(PDF)

Figure S2 **Effect of γ-secretase inhibition on gene programs of interest in SKRC-10 cells.** (**A**) Notch inhibition does not profoundly affect the TGF-β cytostatic gene program as assessed by gene expression analysis of SKRC-10 cells. Isolated and purified RNA from SKRC-10 cells treated with vehicle control (c) or DAPT in 1% FCS for 24 hours was used in oligomer microarray experiments. Data represents mean log_2_ ratios of three independent experiments+95% confidence intervals. (**B**) Notch inhibition leads to downregulation of a large set of genes (* indicates previously described TGF-β target genes) associated with cell migration and invasion as determined by gene expression analysis of SKRC-10 cells. Isolated and purified RNA from SKRC-10 cells treated with vehicle control (c) or DAPT in 1% FCS for 24 hours was used in oligomer microarray experiments. Data represents mean log_2_ ratios of three independent experiments+95% confidence intervals. ***, ** and * indicates statistical significant changes (two-sided Student's t-test, p<0.001, p<0.01 and p<0.05 respectively).
(PDF)

Table S1 **Core TGF-β gene expression signature of 145 genes.**
(PDF)

Table S2 **Multivariate COX regression analyses.**
(PDF)

Table S3 **Q-PCR primer sequences.**
(PDF)
