# Supplementary figures and images for: The Notch and TGF-β Signaling Pathways Contribute to the Aggressiveness of Clear Cell Renal Cell Carcinoma
Source: PLoS One. 2011 Aug 3;6(8):e23057. doi: 10.1371/journal.pone.0023057 (PMC3149633; doi:10.1371/journal.pone.0023057)

Figure S1

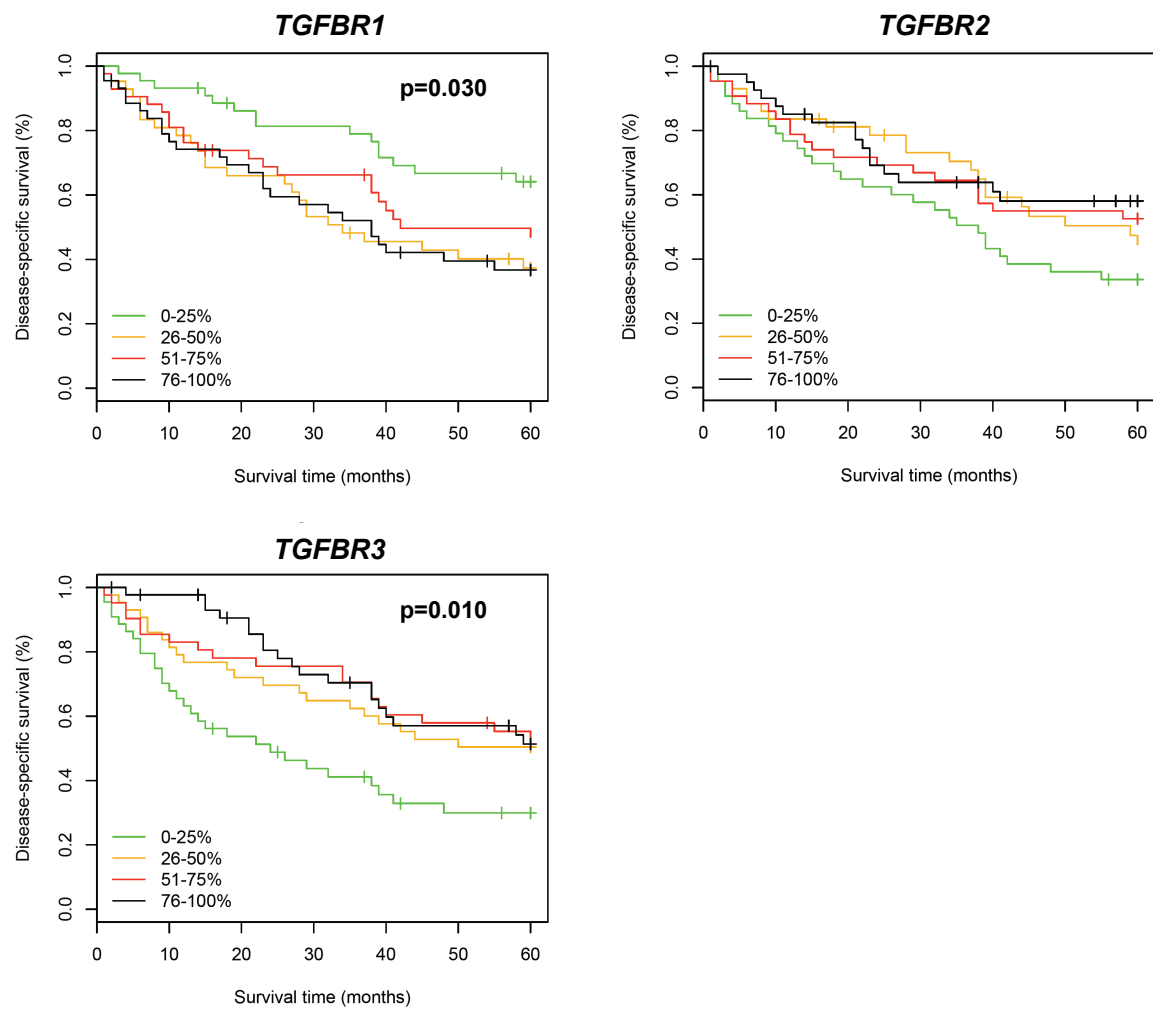

Supplement: Figure S1 — Disease-specific survival of 176 CCRCC patients based on the gene expression of TGFBRs. Kaplan-Meier plots of disease-specific survival of 176 CCRCC patients that were divided into four groups based on the median gene expression values of TGFBR1, TGFBR2, and TGFBR3. Elevated TGFBR1 expression (log-rank, p = 0.030) and decreased (0–25%) TGFBR3 expression (log-rank, p = 0.010) were significantly associated with worse disease-specific survival. (PDF) [file pone.0023057.s001.pdf]

Figure S2

A

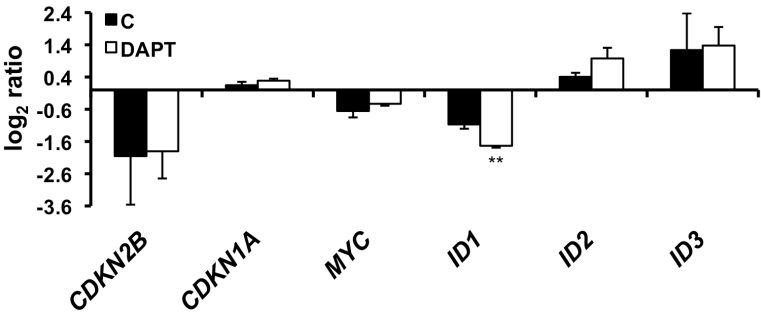

B

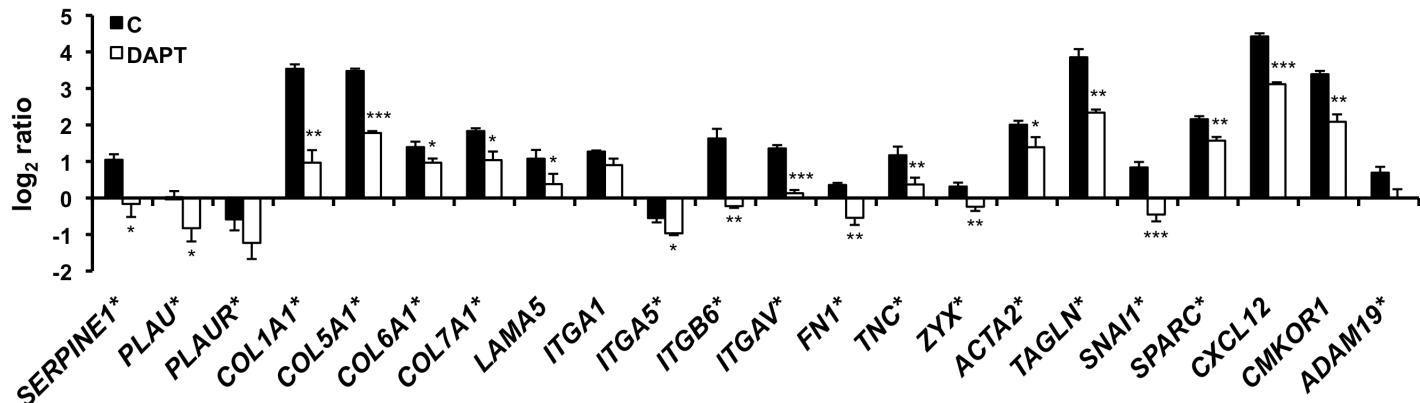

Supplement: Figure S2 — Effect of γ-secretase inhibition on gene programs of interest in SKRC-10 cells. (A) Notch inhibition does not profoundly affect the TGF-β cytostatic gene program as assessed by gene expression analysis of SKRC-10 cells. Isolated and purified RNA from SKRC-10 cells treated with vehicle control (c) or DAPT in 1% FCS for 24 hours was used in oligomer microarray experiments. Data represents mean log2 ratios of three independent experiments+95% confidence intervals. (B) Notch inhibition leads to downregulation of a large set of genes (* indicates previously described TGF-β target genes) associated with cell migration and invasion as determined by gene expression analysis of SKRC-10 cells. Isolated and purified RNA from SKRC-10 cells treated with vehicle control (c) or DAPT in 1% FCS for 24 hours was used in oligomer microarray experiments. Data represents mean log2 ratios of three independent experiments+95% confidence intervals. ***, ** and * indicates statistical significant changes (two-sided Student's t-test, p<0.001, p<0.01 and p<0.05 respectively). (PDF) [file pone.0023057.s002.pdf]
