# Supplementary material for: The Notch and TGF-β Signaling Pathways Contribute to the Aggressiveness of Clear Cell Renal Cell Carcinoma
Source: PLoS One. 2011 Aug 3;6(8):e23057. doi: 10.1371/journal.pone.0023057 (PMC3149633; doi:10.1371/journal.pone.0023057)
Supplement: Table S1 — Core TGF-β gene expression signature of 145 genes. (PDF) [file pone.0023057.s003.pdf]

Table S1

Order within gene set	Symbol	Rank within gene set	Reference	Regulation
1	*COL1A1*	2	1	UP
2	*SPHK1*	15	1	UP
3	*SNAI1*	22	1	UP
4	*TMEPAI*	24	1	UP
5	*JUNB*	30	1	UP
6	*FSTL3*	36	1	UP
7	*SERPINE1*	43	1	UP
8	*LMCD1*	46	1	UP
9	*SGK*	71	1	UP
10	*ARFGAP1*	87	1	UP
11	*NDST1*	116	1	UP
12	*JAG1*	131	1	UP
13	*TUFT1*	141	1	UP
14	*SKIL*	166	1	UP
15	*ETS2*	216	1	UP
16	*COL4A1*	217	1	UP
17	*GADD45B*	273	1	UP
18	*TPM1*	369	1	UP
19	*RHOB*	380	1	UP
20	*SSBP3*	477	1	UP
21	*PPP1R13L*	478	1	UP
22	*COL4A2*	540	1	UP
23	*AMIGO2*	614	1	UP
24	*TBC1D2B*	843	1	UP
25	*HMOX1*	970	1	UP
26	*C18orf25*	1003	1	UP
27	*PDGFA*	1033	1	UP
28	*ARL6IP2*	1183	1	UP
29	*IL11*	1272	1	UP
30	*ELK3*	1276	1	UP
31	*CTGF*	1307	1	UP
1	*PDLIM7*	13	2	UP
2	*CXCL12*	21	2	UP
3	*SNAI1*	22	2	UP
4	*TMEPAI*	24	2	UP
5	*SERPINE1*	43	2	UP
6	*SGK*	71	2	UP
7	*TFPI2*	93	2	UP
8	*JAG1*	131	2	UP
9	*SKIL*	166	2	UP
10	*ACSL3*	267	2	UP
11	*GADD45B*	273	2	UP
12	*LGMN*	320	2	UP
13	*IER3*	342	2	UP
14	*RHOB*	380	2	UP
15	*SLC29A1*	466	2	UP
16	*HMGA2*	500	2	UP
17	*SLC20A1*	727	2	UP
18	*HCRT*	863	2	UP
19	*ZSWIM4*	932	2	UP
20	*SERINC2*	947	2	UP
21	*PDGFA*	1033	2	UP
22	*HCN2*	1086	2	UP
23	*CTGF*	1307	2	UP
24	*IGFBP3*	1344	2	UP

25	*SLC25A37*	1883	2	UP
26	*CDA*	1981	2	UP
27	*LRP1*	2106	2	UP
28	*SERINC5*	2111	2	UP
29	*ALAS1*	2162	2	UP
30	*RELB*	2393	2	UP
31	*NDEL1*	2521	2	UP
32	*NGFB*	2587	2	UP
33	*TGIF*	3033	2	UP
34	*LPGAT1*	3056	2	UP
35	*DHX32*	3247	2	UP
36	*TGFBR1*	3284	2	UP
37	*ACVR1*	3313	2	UP
38	*CSNK1E*	3397	2	UP
39	*ARID3A*	3433	2	UP
40	*BMPR2*	3479	2	UP
1	*SERPINE1*	43	3	UP
2	*SEMA3F*	51	3	UP
3	*TNC*	109	3	UP
4	*FN1*	122	3	UP
5	*MMP14*	136	3	UP
6	*EPHB2*	145	3	UP
7	*SPARC*	207	3	UP
8	*ZYX*	243	3	UP
9	*IGFBP5*	310	3	UP
10	*THBS1*	313	3	UP
11	*RHOB*	380	3	UP
12	*ITGA5*	410	3	UP
13	*MMP1*	413	3	UP
14	*COL6A1*	431	3	UP
15	*ITGB3*	629	3	UP
16	*MMP2*	826	3	UP
17	*EPHA2*	1039	3	UP
18	*RHOG*	1259	3	UP
19	*ARHGAP1*	1275	3	UP
20	*ARHGDIA*	1301	3	UP
21	*WNT2B*	1330	3	UP
22	*IGFBP3*	1344	3	UP
23	*MMP11*	1591	3	UP
24	*EPHB4*	1948	3	UP
25	*TNFRSF1A*	2096	3	UP
26	*LRP1*	2106	3	UP
27	*CTNNA1*	2469	3	UP
28	*ICAM1*	2543	3	UP
29	*COL6A3*	2700	3	UP
1	*OLIG2*	30	1	DOWN
2	*CEBPD*	87	1	DOWN
3	*RAI2*	106	1	DOWN
4	*CITED2*	259	1	DOWN
5	*DOPEY1*	310	1	DOWN
6	*AVPI1*	534	1	DOWN
7	*MYBL1*	734	1	DOWN
8	*AHI1*	832	1	DOWN
9	*ZNF395*	1140	1	DOWN
10	*ZNF318*	1485	1	DOWN
11	*MYC*	1516	1	DOWN
12	*DDIT4*	1537	1	DOWN
13	*IL5*	1820	1	DOWN
14	*FAT4*	1907	1	DOWN
15	*ATF7IP*	2224	1	DOWN

1	*IGFBP4*	3	2	DOWN
2	*HABP2*	20	2	DOWN
3	*ALDH1A1*	82	2	DOWN
4	*MAL2*	135	2	DOWN
5	*ALDH3A2*	248	2	DOWN
6	*ERRFI1*	346	2	DOWN
7	*ABCC3*	358	2	DOWN
8	*UBE2L6*	366	2	DOWN
9	*OTUD1*	524	2	DOWN
10	*TGM1*	829	2	DOWN
11	*FARSLB*	871	2	DOWN
12	*CXCL1*	958	2	DOWN
13	*KCTD3*	1063	2	DOWN
14	*NAB1*	1156	2	DOWN
15	*DUSP3*	1204	2	DOWN
16	*VIM*	1214	2	DOWN
17	*SERPIND1*	1266	2	DOWN
18	*NBEAL1*	1379	2	DOWN
19	*GSS*	1411	2	DOWN
20	*NFKBIA*	1514	2	DOWN
21	*BCL3*	1552	2	DOWN
22	*ITGA6*	1560	2	DOWN
23	*SCP2*	1598	2	DOWN
24	*DUS1L*	1640	2	DOWN
25	*ERGIC1*	1657	2	DOWN
26	*CBS*	1743	2	DOWN
27	*DHCR24*	1815	2	DOWN
28	*IKBKE*	1902	2	DOWN
29	*CHMP4C*	1916	2	DOWN
30	*ARHGAP6*	1967	2	DOWN
31	*GPD1*	2061	2	DOWN
32	*IDH1*	2158	2	DOWN
33	*PLAT*	2212	2	DOWN
34	*SEPP1*	2222	2	DOWN
35	*LGALS3*	2261	2	DOWN
36	*IMPACT*	2506	2	DOWN
37	*ENTPD5*	2815	2	DOWN
38	*TMEM97*	2850	2	DOWN
39	*SORBS2*	2980	2	DOWN
40	*GRHPR*	3039	2	DOWN
41	*TSC22D2*	3226	2	DOWN
42	*LRRC35*	3320	2	DOWN
43	*NFE2L2*	3385	2	DOWN
44	*NET1*	3445	2	DOWN

References

1. Padua D, Zhang XH, Wang Q, Nadal C, Gerald WL, Gomis RR et al. (2008) TGF-beta primes breast tumors for lung metastasis seeding through angiopoietin-like 4. Cell 133(1): 66-77.

2. Coulouarn C, Factor VM, Thorgeirsson SS (2008) Transforming growth factor-beta gene expression signature in mouse hepatocytes predicts clinical outcome in human cancer. Hepatology 47(6): 2059-67.

3. Verrecchia F, Chu ML, Mauviel A (2001) Identification of novel TGF-beta /Smad gene targets in dermal fibroblasts using a combined cDNA microarray/promoter trans-activation approach. J Biol Chem 276(20): 17058-62.
