# Supplementary material for: The Notch and TGF-β Signaling Pathways Contribute to the Aggressiveness of Clear Cell Renal Cell Carcinoma
Source: PLoS One. 2011 Aug 3;6(8):e23057. doi: 10.1371/journal.pone.0023057 (PMC3149633; doi:10.1371/journal.pone.0023057)
Supplement: Table S2 — Multivariate COX regression analyses. (PDF) [file pone.0023057.s004.pdf]

Table S2

Parameters	Univariate Analysis	Multivariate Analysis	Hazard Ratio	Lower 0.95	Upper 0.05
**TGF-β Pathway Score**	0.00129	0.00433	4.036	1.548	10.527
**Stage**	<2e-16	< 2e-16	3.652	2.731	4.884
**Grade**	2.06E-05	0.0489	1.383	1.002	1.909
**Performance Status**	6.80E-07	0.00524	1.329	1.088	1.622
