# Supplementary material for: The Notch and TGF-β Signaling Pathways Contribute to the Aggressiveness of Clear Cell Renal Cell Carcinoma
Source: PLoS One. 2011 Aug 3;6(8):e23057. doi: 10.1371/journal.pone.0023057 (PMC3149633; doi:10.1371/journal.pone.0023057)
Supplement: Table S3 — Q-PCR primer sequences. (PDF) [file pone.0023057.s005.pdf]

Table S3

Gene	Forward (5´- 3´)	Reverse (5´- 3´)
SDHA	TGGGAACAAGAGGGCATCTG	CCACCACTGCATCAAATTCATG
YWHAZ	ACTTTTGGTACATTGTGGCTTCAA	CCGCCAGGACAAACCAGTAT
UBC	ATTTGGGTCGCGGTTCTT	TGCCTTGACATTCTCGATGGT
HES1	AGCGGGCGCAGATGAC	CGTTCATGCACTCGCTGAA
SERPINE1	CAGACCAAGAGCCTCTCCAC	ATCACTTGGCCCATGAAAAG
SKIL	CACCCCAGCTACTACTTATAC	TTGCCTCTGTCTTTGTGAGC
JUNB	CACGACGACTCTTACGCAGC	GACCCTTGAGACCCCGATAA
